# RRS1 gene expression involved in the progression of papillary thyroid carcinoma

**DOI:** 10.1186/s12935-018-0519-x

**Published:** 2018-02-13

**Authors:** Feng Chen, Yaqiong Jin, Lin Feng, Jie Zhang, Jun Tai, Jin Shi, Yongbo Yu, Jie Lu, Shengcai Wang, Xin Li, Ping Chu, Shujing Han, Shujun Cheng, Yongli Guo, Xin Ni

**Affiliations:** 1 Beijing Key Laboratory for Pediatric Diseases of Otolaryngology, Head and Neck Surgery, MOE Key Laboratory of Major Diseases in Children, Beijing Pediatric Research Institute, Beijing Children’s Hospital, Capital Medical University, National Center for Children’s Health, Beijing, China; 2 Department of Otolaryngology, Head and Neck Surgery, Beijing Children’s Hospital, Capital Medical University, National Center for Children’s Health, No.56 Nanlishi Rd., Beijing, 100045 China; 3 Biobank for Clinical Data and Samples in Pediatric, Beijing Pediatric Research Institute, Beijing Children’s Hospital, Capital Medical University, National Center for Children’s Health, Beijing, China; 4 State Key Laboratory of Molecular Oncology, Department of Etiology and Carcinogenesis, Peking Union Medical College and Cancer Institute (Hospital), Chinese Academy of Medical Sciences, Beijing, China; 5 Department of Biochemistry and Molecular Biology, Peking University Health Science Center, Beijing, China

**Keywords:** Papillary thyroid carcinoma, RRS1, Cancer progression

## Abstract

**Background:**

Papillary thyroid carcinoma (PTC) is one of the most frequent malignancies of the endocrine system, whose mechanisms of pathogenesis, progression and prognosis are still far from being clearly elucidated. Despite an increasing body of evidences highlights ribosome biogenesis regulator homolog (RRS1) as a ribosome biogenesis protein in yeast and plants, little is known about human RRS1 function.

**Methods:**

Proliferation, cell cycle and apoptosis of PTC cells were assessed following the knockdown of RRS1 expression though MTT, colony formation assay, and flow cytometry. Then, transcriptome profiling was conducted to explore pathway changes after RRS1 silencing in PTC cells. Receiver operating characteristic curve and Youden’s index were performed in twenty-four thyroid carcinoma samples to assess their potential clinical diagnostic value.

**Results:**

Firstly, we found that silencing RRS1 significantly reduced cell proliferation, inhibited cell cycle, and promoted apoptosis in PTC cell line. The result also showed that knock-down of RRS1 could up-regulate genes involving apoptosis and metabolism, while, down-regulate genes relative to cell proliferation and blood vessel development. Notably, the present study confirmed the diagnostic value of RRS1 for thyroid carcinoma in both children and adults.

**Conclusions:**

In conclusion, these data afford a comprehensive view of a novel function of human RRS1 by promoting cell proliferation and could be a potential indicator for papillary thyroid carcinoma.

**Electronic supplementary material:**

The online version of this article (10.1186/s12935-018-0519-x) contains supplementary material, which is available to authorized users.

## Background

Thyroid cancer is the most common endocrine cancer [1]. The incidence of thyroid cancer is increasing faster than any other cancer in China, with expected 90,000 patients to be diagnosed in 2015 [2]. Thyroid cancer mainly comprises differentiated thyroid cancer (DTC) and medullary thyroid cancer (MTC). Therein, a large proportion (78–90%) of DTC is papillary thyroid carcinoma (PTC) in adults [3, 4]. Similar with adults cases, PTC accounts for 90% or more of thyroid cancer cases in childhood [5]. However, other types of thyroid cancer, such as follicular thyroid cancer, medullary thyroid cancer, and anaplastic thyroid carcinoma, are rare in young patients [6]. Although efforts have been done in tumorigenesis, little is known about PTC. This type of tumor occurs mostly in women; sex hormones may play a role in the pathogenesis [7]. In children, especially in those under 5 years old, the major risk factor for developing PTC is radiation exposure [6, 8]. Although PTC has a favorable prognosis, certain cases exhibit aggressive clinical characteristics, such as lymph node and distance metastasis, and poorer prognosis. Once regional nodal metastases or aerodigestive invasion is discovered, radioiodine may be employed [3]. Radioiodine treatment can significantly increase event-free survival of PTC patients. Lower dose of radioiodine applied in therapy do not affect patients’ daily life. However, patients with distance metastases, under radioactive iodine treatment (≥ 150 mCi), are at risk for poor life quality due to physical pain, lower vitality, bad mental health, and physical/emotional problems [9]. Therefore, development of prognostic factors is important to early diagnosis of PTC, following establishment of therapeutic strategies. Many laboratory and clinical studies have been carried out for investigation of new markers to predict prognostic of PTC. For instance, clinical markers, such as extra thyroidal extension and lymph node metastasis (LNM), have been widely used to characterize persistent/recurrent PTC at initial diagnosis.

Beside clinical indicators, molecular markers are also very important in prognosis of PTC. In this regard, the expression of galectin-3, thyroid peroxidase and keratin 19 are all widely used in diagnosis of PTC for adults [10]. However, these indicators are lack of evaluation in pediatric PTC samples. Several studies had showed different molecular mechanism of PTC tumorigenesis in children and adults. For instance, 2015 American Thyroid Association (ATA) Management guidelines for children points out that BRAF mutations are the most common abnormality in adult PTC (36–83%) [6], but rare in children PTC. In contrast, RET/PTC rearrangements are more common in PTC from children than that in adults (45% vs. 35%) [6, 11, 12]. Nonetheless, the clinical applications of these molecular markers are still limited due to low sensitivity and specificity [13]. Therefore, hypersensitive and specific diagnostic test for both children and adults are needed to be developed.

RRS1 was found as a regulatory protein for ribosome biogenesis in yeast [14]. So far, the function of human RRS1 is far from fully understood. Gambe et al. found that RRS1 protein contributed to chromosome congression in HeLa cells [15]. Moreover, Carnemolla et al. reported that RRS1 involved in ER stress which was the early event of Huntington disease [16]. However, the role of human RRS1 in cancer is still unknown. Rapid cell proliferation is a biological feature of cancer, which accompanies with enlarged nucleolus. The available evidences indicate that the nucleolar changes in morphology or function are the consequence of increased demand for ribosome biogenesis. PTC has several significant alterations which are observed both in the fine structure and in the number of nucleoli [17, 18]. These studies raise the possibility of the interference of a ribosome biogenesis thus inducing PTC pathogenesis. Consistent with its function in yeast, human RRS1 ortholog was identified in a proteome analysis of nucleolar extracts [15, 19]. We thus assumed that RRS1 may play essential role in PTC proliferation.

Here we presumed that dysregulation of RRS1 in thyroid cancer played an important role in cell proliferation and thyroid carcinoma progression. Our results support a novel function of RRS1 by promoting thyroid carcinoma cells proliferation, which could be a potential indicator of PTC in both children and adults.

## Methods and patients

### Cell lines and culture

Human papillary thyroid carcinoma cell line TPC-1 was obtain from American Type Culture Collection. Cells were seeded in DMEM (Gibico) with 10% fetal bovine serum (Life Technologies, Inc., Gaithersburg, MD), 100 mg/ml streptomycin and 100 units/ml penicillin [20]. Cells were grown at 37 °C under 5% CO_2_. The TPC-1 cells had been tested within 3 months before the conduction of this study for morphologic features of thyroid carcinoma cells by GENECHEM co. (Shanghai, China).

### Real-time PCR

Total RNA was isolated with Trizol reagent (Invitrogen) according to the manufacturer’s protocol for total RNA isolation. And then the RNA (2 μg) was subjected to reverse transcription by using M-MuLV reverse transcriptase (Fermentas). Real-time PCR amplification was performed using primers pairs for RRS1: (sense) 5′-CCCTACCGGACACCAGAGTAA-3′, (anti-sense) 5′-CCGAAAAGGGGTTGAAACTTCC-3′; GAPDH: (sense) 5′-TGACTTCAACAGCGACACCCA-3′, (anti-sense) 5′-CACCCTGTTGCTGTAGCCAAA-3′. And real-time PCR was performed using a SYBR Green/Fluorescein PCR master mix (Applied Biosystems) with a thermal profile of 95 °C for 5 min followed by 40 cycles of 94 °C for 20 s, 60 °C for 1 min. PCR reactions in triplicate were carried out by using an ABI ViiA7™ Real-time PCR system (Life Technology).

### Western blotting

Equal amounts (30 μg) of the total protein of the cells were subjected to 10% SDS-PAGE, blotted onto nitrocellulose membranes (Pall Life Sciences). Membranes were then blocked at room temperature for 1 h and probed with specific antibodies (1:1000 to 1:10,000) for RRS1 (ab117846, abcam) and GAPDH (sc-32233, Santa-Cruz) at 4 °C for overnight. The membranes were further probed with the secondary antibody, HRP-labeled goat anti-rabbit IgG (sc-2004, Santa-Cruz) or goat anti-mouse IgG (sc-2005, Santa-Cruz). After the ECL chemiluminescence kit (Amersham Biosciences), bands were visualized by ChemiDoc XRS + (Bio-Rad).

### Short hairpin RNA constructs and antibodies

For RRS1 short hairpin RNA (shRNA), second-generation lentiviruses expressing shRNA-RRS1 and shRNA-Negative control (shRNA-NC) were used. Briefly, 293T cells were transfected with pLSLPw, TRIPZ, and GIPZ constructs along with packaging plasmids, pVSVG, and pLV-CMV-delta 8.2 by using lipofectamine. Virus-containing supernatants were collected at 48 and 72 h, and thyroid carcinoma cells were transduced in the presence of 8 mg/ml polybrene (Sigma).

### Oligonucleotide microarray data analysis

Total RNA was isolated using RNeasy Kit (Qiagen) from TPC-1 cells infected with RRS1 shRNA or NC shRNA lentiviruses. Affymetrix GeneChip^®^ PrimeView™ Human Gene Expression Array was performed in thyroid carcinoma cells upon knock-down of RRS1 for 4 days in triplicate. Differential expression of genes were identified by RRS1 silencing (*P*-values 0.05), ANOVA (*P*-value 0.05), and absolute value (twofold change). Logarithm of fluorescence intensity ratio was represented by fold change, and log2 ratios ≤ − 1.0 or log2 ratios ≥ 1.0 means two fold changes. Genes were ranked regarding to average expression changes upon RRS1 knock-down. Network function analysis was performed using IPA^®^ (Interactive pathway analysis of complex omics data) software. Gene ontology (GO) analysis of differentially expressed genes was performed using DAVID (https://david.ncifcrf.gov/) [21]. Gene Set Enrichment Analysis (GSEA) was then performed using GSEA software v2.0.14 [22]. Default parameters were used and gene sets that met the false discovery rate (FDR) 0.25 criterion were ranked by nominal *P* value.

### Flow cytometry analysis

shRNA-NC or shRNA-RRS1 Cells were grown in 6-well plates for 2 days. Then, cells were trypsinized by 0.25% trypsin (EDTA free). Harvested cells were washed twice in ice-cold PBS and fixed in 70% ice-cold ethanol for overnight at 4 °C. Cells were washed twice in PBS and digested with RNase A (50 μ/ml) at 37 °C for 30 min. Cells were then resuspended in 500 μl propidium iodide (10 μg/ml; Sigma) 10 min prior to flow cytometry analysis. A FACScan (Becton–Dickinson) was used to analyze cellular DNA content. For cell cycle analysis, computer programs CELLQuest and ModFit LT 2.0ep for power were used.

### Apoptosis assay

Apoptosis assays were performed using Annexin V- APC Apoptosis Assay Kit (Biosea, China). After trypsinized, cells were collected and washed with ice-cold PBS twice, and re-suspended at a concentration of 0.5–1.0 × 10^6^ cells/ml in binding buffer. Cells (100 μl) were supplemented with 5 μl of fluorochrome-conjugated Annexin V (1 μg/ml). The mixture was incubated at room temperature for 15 min in the dark, and then 200 μl binding buffer was added. Cells were analyzed by flow cytometry within 30 min of staining.

### Colony formation assay

The effect of RRS1 knockdown on colony formation ability of the TPC1 cells was detect by plate colony formation assay. Forty-eight hours after short hairpin RNA affection, cells were plated at limiting dilution (800 cell per well) in 6-well format in triplicate. Cells were fed twice weekly until control colonies began to merge. Samples were fixed with 4% formaldehyde and stained with Geimsa. Plates were scans for the number of colonies under CloneSelect Imager (Molecular Devices).

### Clinical patients

The study set consisted of 24 patients (14 adults and 10 children) who were diagnosed with papillary thyroid carcinoma. Twenty-four carcinoma tissues and 14 pericarcinous tissues (paired with 14 carcinoma tissues), totally 38 samples, were collected (Table 1). Ten children patients were recruited from January 2013 to December 2015, at the Beijing Children’s Hospital, Capital Medical University. Fourteen adult patients were recruited at Beijing Tongren Hospital, Capital Medical University, from January 2014 to January 2015. All diagnoses of papillary thyroid carcinoma were made by pathological examinations of resected samples from surgery or needle biopsy specimens. Disease staging for each patient was determined by at least two pathologists according to Union for International Cancer Control (UICC) Staging System. Sample were drawn from each participant together with pathological examination immediately immerged into RNAlater^®^ within 30 min, and then they were stored at − 80 °C.

**Table 1:** Frequency and distribution of clinical characteristics

Variable	Tumor (N = 24)		Normal (N = 14)	
	No.	(%)	No.	(%)
Gender				
Male	5	20.83	3	21.43
Female	19	79.17	11	84.62
Age (years)				
< 18	10	41.67	3	23.08
≥ 18	14	58.33	11	76.92
Tumor stage^a^				
I	15	62.50		
II	9	37.50		
Regional lymph node^a^				
N0	4	16.67		
N1	19	79.17		
N2	1	4.16		
Metastasis^a^				
M0	23	95.83		
M1	1	4.17		

^a^According to Union for International Cancer Control (UICC) Staging System

### Statistical analysis

For Real-time PCR, MTT assay, plate colony formation, apoptosis assay and Flow Cytometry, values were obtained from at least 3 independent experiments in PTC-1 cell line. Data were analyzed using two-tailed Student T-test, and a value of *P* < 0.05 was considered to be statistically significant. For comparing of RRS1 expression between carcinoma tissues and pericarcinous tissues, unpaired T-test or paired T-test (for paired tissue) was performed. ROC curve, the area under the curve (AUC) and Youden’s index were performed to further evaluation of the diagnostic value of RRS1 in PTC. These statistical analyses above were conducted by using GraphPad Prism 6.0 software (GraphPad Software, Inc).

## Results

### Down-regulation of RRS1 inhibits papillary thyroid carcinoma cells proliferation

**Fig. 1:**
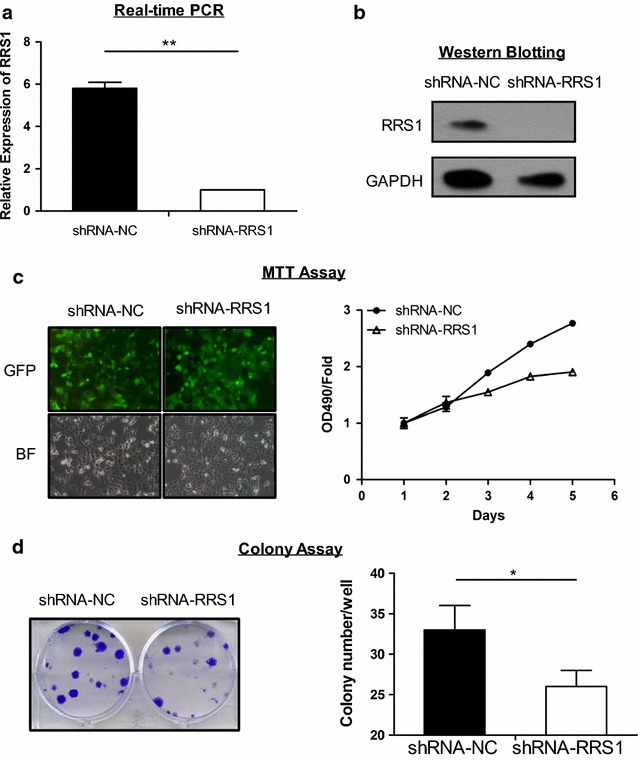
Knock-down of RRS1 inhibited TPC-1 cells proliferation. **a**, **b** Real-time PCR and Western blotting were performed to detect the efficiency of RRS1 knock-down. **c** Proliferation of TPC-1 cells was expressing shRNA targeted against negative and RRS1. Plotted data were mean ± s.e.m. of three independent experiments. *P < 0.05 from student T-test. **d** Colony formation assays of TPC-1 cells expressing shRNAs were quantified for relative colony numbers of three independent experiments. *P < 0.05 from student T-test. Three independent experiments were performed

To evaluate the role of RRS1 in progression of papillary thyroid carcinoma, lentiviral shRNA-mediated silencing was employed. As shown in Fig. 1a and b, endogenous mRNA and protein of RRS1 was reduced after transfection with shRNA-RRS1 or controls in TPC-1 cell line. In TPC-1 cell line, knock-down of RRS1 induced with GFP-lentiviral shRNA was visualized by fluorescent microscopy for GFP (Fig. 1c). Then, MTT assay was performed to monitor cell proliferation, showing that RRS1 knock-down suppressed cell proliferation from day 2 to day 5 (Fig. 1c). To investigate the oncogenic potential of RRS1 in thyroid carcinoma, colony formation assays were performed in the 6-well cell culture plates. The results showed that compared with shRNA-NC, RRS1 silencing inhibited colony formation in TPC-1 cells (Fig. 1d). These data indicated that RRS1 expression contributed to cells proliferation in papillary thyroid carcinoma.

### Down-regulation of RRS1 induces apoptosis in papillary thyroid carcinoma cells

**Fig. 2:**
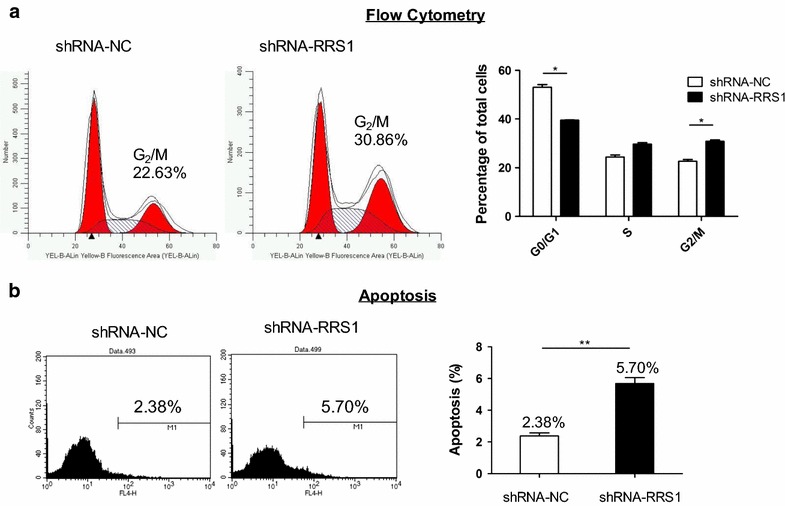
Knock-down of RRS1 induced G_2_/M phase arrest and apoptosis in TPC-1 cells. **a** Left: one representative flow cytometry analysis of the cell cycle; right: histograms showed the percentage of cell subpopulations in G_1_, S, and G_2_/M phases. Data represent mean ± SE (n = 3). **b** Left: one representative flow cytometry analysis of the Annexin-V; right: histograms showed the percentage of cell subpopulations in M1 gate (Annexin-V positive cells). Data represent mean ± SE (n = 3). Three independent experiments were performed. *P value < 0.1, **P value < 0.01, ***P value < 0.001

Furthermore, to illustrate the role of RRS1 in cell cycle progression, flow cytometry was performed to detect cycle progression with knock-down of RRS1 in TPC-1 cells. Introducing shRNA-RRS1 into TPC-1 cells for 48 h caused an increasing of G_2_/M phase and S phase subpopulations from 22.63 to 30.86% (*P* < 0.05) and from 24.35 to 29.68% (*P* > 0.05), respectively, while G_1_ subpopulation was decreased from 53.02 to 39.46% (*P* < 0.05) simultaneously (Fig. 2a). These data showed that down-regulation of RRS1 could activate G_2_/M phase checkpoint, thus inhibited TPC-1 cells proliferation.

To examine the roles of RRS1 upon lentiviral-mediated silencing in cell apoptosis, Annexin V staining was performed followed with flow cytometry (Fig. 2b). Silencing RRS1 increased apoptosis from 2.38 to 5.70% (*P* < 0.001) compared with scramble lentiviral (shRNA-NC). The results suggested that inhibitory proliferation and apoptosis of thyroid carcinoma cells could be induced by knock-down of RRS1.

### Down-regulation of RRS1 inhibits major oncogenic pathways and induces apoptosis pathways

**Fig. 3:**
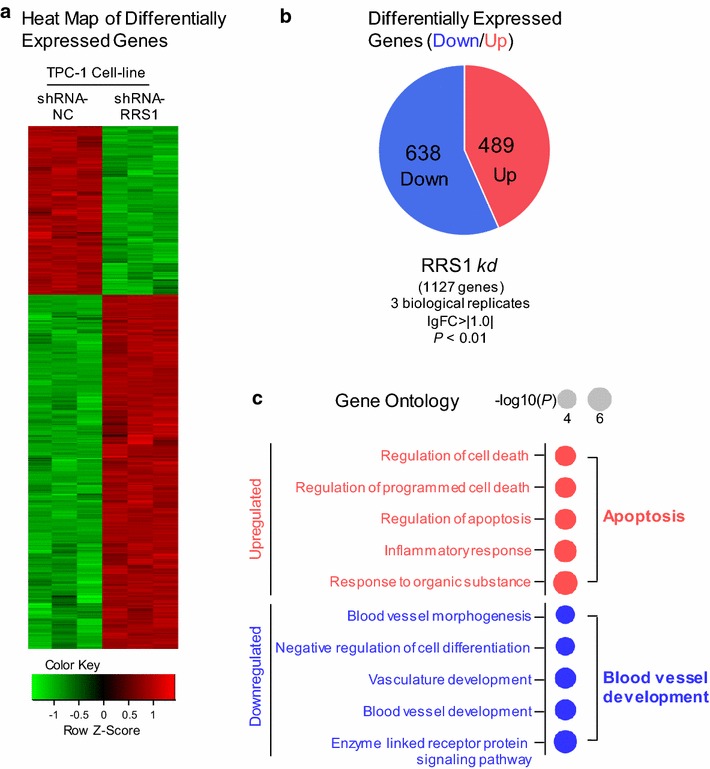
Microarray assay of RRS1 knock-down cells revealed dramatic alterations of the cancer cell transcriptome. **a** Heatmap of the 1127 genes altered in both negative control and RRS1 silencing, showing up- (red) and down- (green) regulated genes. **b** Pie chart showing 489 up- (red) and 683 down- (blue) regulated genes as calculated by both DESeq2 and edgeR algorithms with cutoffs as indicated. **c** Dot plots of IPA diseases and functions enriched in both knock-downs were shown in dots scaled by − log (*P*)

To unveil changes in gene expression associated with phenotype alterations involved with RRS1 in thyroid carcinoma cells, gene expression profiling (Affymetrix 901838) was performed after RRS1 silencing in TPC-1 cells. Clustered heat map of the differentially expressed genes with RRS1 silencing was showed in Fig. 3a. Standard selection criteria to identify differentially expressed genes are as follows: log2 ratios ≥ 1.0 means up-regulated, and log2 ratios ≤ −1.0 represents down-regulation (*P* < 0.05, Additional file 1: Fig. S1). The data showed that silencing RRS1 could up-regulate 489 genes, and down-regulate 638 genes respectively (Fig. 3b, Additional file 2: Table S1).

Gene ontology (GO) was performed to further explore cell processes and precise pathways involved with RRS1. Upregulated GO terms upon RRS1 silencing were enriched in cell death/apoptosis (Fig. 3c), while downregulated GO terms were involved in blood vessel development (Fig. 3c).

**Fig. 4:**
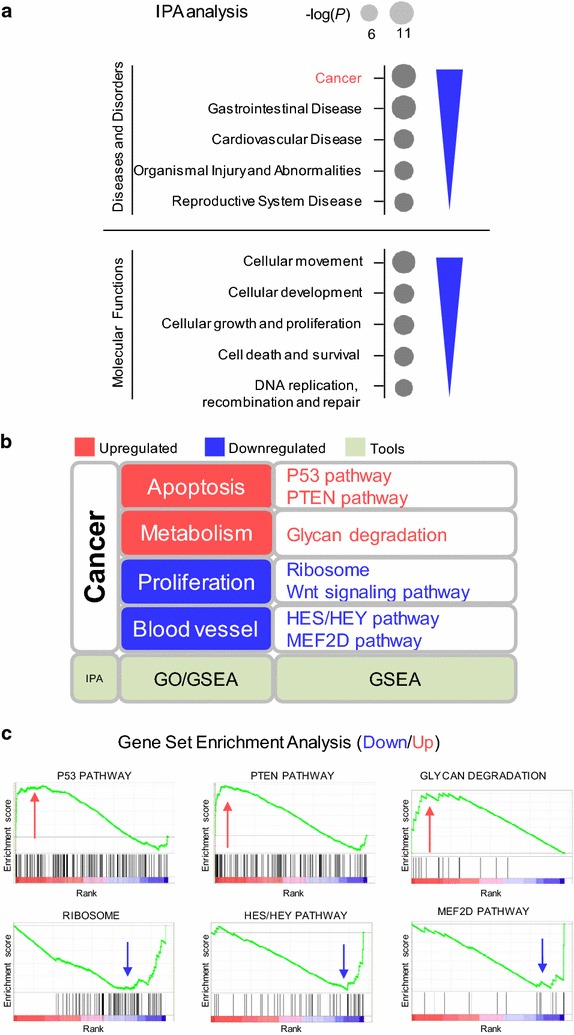
Bioinformatics analyze of RRS1 knock-down cells revealed dramatic alterations of transcriptome in the thyroid cancer cells. **a** Dot plots of gene ontology terms enriched on RRS1 knock-down upregulated genes (top panel, red) and downregulated genes (bottom panel, blue) were shown in dots scaled by − log10 (*P*). **b** Example gene set enrichment analysis of genes altered in RRS1 knock-downs. Top panel: Ribosome related genes had a significant negative normalized enrichment score (NES) upon RRS1 knock-down. Middle-up panel: HES/HEY pathway related genes had a significant NES upon RRS1 knock-down. Middle-down panel: MEF2D pathway related genes had a significant NES upon RRS1 knock-down. Bottom panel: Glycan degradation gene set had a significant positive NES upon knock-down of RRS1. **c** Summary of microarray assay by multiple bioinformatics tool

Subsequently, ingenuity pathway analysis (IPA) was used to probe enriched pathways of the differentially expressed genes. IPA showed that cancer and cellular movement were the most highly enriched terms in “Diseases and Disorders” and “Molecular Functions”, respectively (Fig. 4a). To further analyze this data set, we performed gene set enrichment analysis with candidate sets (Fig. 4b). RRS1 silencing suppressed apoptosis pathways, including P53 pathway, PTEN pathway (*P* < 0.05, FDR q < 0.25). Meanwhile, genes expression involved in Glycan degradation (*P* < 0.05, FDR q < 0.25), which related to metabolism, were downregulated in the knockdowns. While RRS1 silencing was enriched in cell proliferation, such as ribosome biogenesis, Wnt signaling pathway, TGF-β signaling pathway, ILK pathway and RHO pathway, (*P* < 0.05, FDR q < 0.25). And pathways for blood vessel development were also downregulated in the knockdowns, like HES/HEY pathway and MEF2D pathway (*P* < 0.05, FDR q < 0.25, Fig. 4c, Additional file 1: Fig. S2). These enrichments analysis supported the hypothesis that RRS1 is necessary to maintain identity of thyroid cancer cell.

### Diagnostic value of RRS1 for thyroid carcinoma among children and adults

Twenty-four thyroid carcinoma tissues and 14 pericarcinous tissues were collected for further investigation. As shown in Table 1, the distribution of gender and age has no significant differences (Chi square statistic, gender *P* = 0.97, age *P* = 0.204). Among tumors, 14 out of 24 (58.33%) were from adults (≥ 18 years), and 10 (41.67%) were from children. According to UICC Staging System, most of patients were in lower stage (I/II, 15 and 9 respectively) and regional lymph node metastasis (N1/N2, 19 and 1 respectively), and one patient of distance metastasis.

**Fig. 5:**
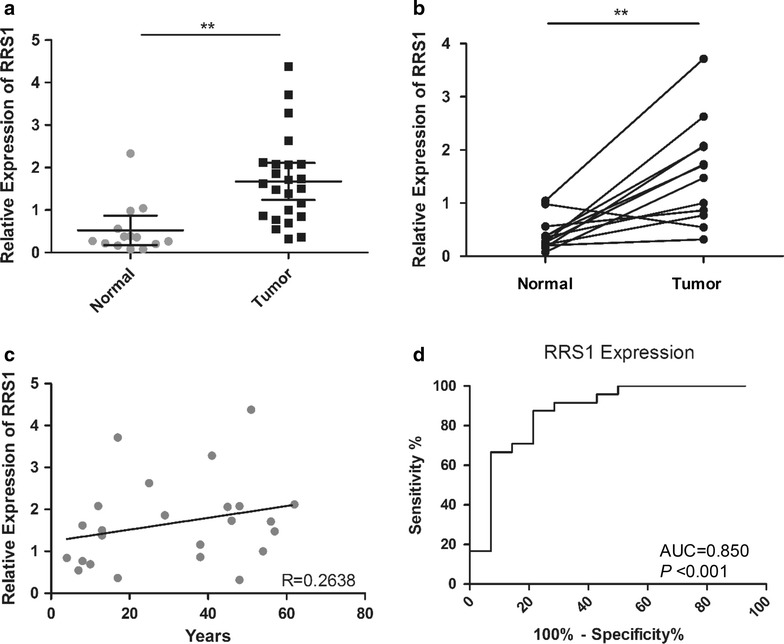
Real-time PCR detected the difference expression and diagnostic value of RRS1 for thyroid carcinoma in children and adults. **a** Expression of RRS1 in PTC (mean = 1.51, n = 24) is higher than that in pericarcinous tissues (mean = 0.54, n = 14, unpaired t-test, *P* < 0.01). **b** Real-time PCR detects RRS1 expression in 12 paired carcinoma and pericarcinous tissues (paired t-test, *P* < 0.01). **c** Pearson correlation coefficient measures the relationship between RRS1 expression and age in PTC tumors (R = 0.2638). **d** Receiver operating characteristic (ROC) curve of 22 PTC tissues and 12 pericarcinous tissues (AUC is 0.850, *P* < 0.0001). *P value < 0.1, **P value < 0.01, ***P value < 0.001

To investigate whether RRS1 differentially expressed in thyroid carcinoma, we detected the expression of RRS1 in total 38 thyroid samples (24 thyroid carcinoma; 14 pericarcinous tissues). The results showed that the expression of RRS1 in PTC (1.51, 95% CI 1.13–1.89) were 2.80-fold higher than that in pericarcinous tissues (0.54, 95% CI 0.17–0.92; unpaired t-test *P* < 0.01, Fig. 5a). To further analysis the difference, 14 paired tissues were employed, showing that RRS1 was significantly overexpressed in thyroid carcinoma other than in paired pericarcinous tissues (paired t-test, *P* < 0.01, Fig. 5b).

In addition, relative expression levels of RRS1 were 1.35, 1.96 and 1.87 in under 18 years (mean 10 years), under 45 years (mean 34 years) and above 45 (mean 52 years) groups respectively (Additional file 1: Fig. S3), suggesting that RRS1 expression was positively correlated with age of onset (R = 0.2638, Fig. 5c). Further, the diagnostic value of RRS1 expression in thyroid carcinoma was evaluated by Receiver Operating Characteristic (ROC) curve analysis and Youden’s index. In total samples of children and adults (24 thyroid carcinoma; 14 pericarcinous tissues), we found that the area under curve (AUC) was 0.850 (95% CI 0.701–0.998, *P* < 0.0001, Fig. 5d, Table 2), and Youden’s index was 0.633 (Table 2). Besides, we found that AUC in adults (0.871, 95% CI 0.718–1.025, *P* < 0.001, Table 2) was higher than that in children (0.823, 95% CI 0.649–0.997, *P* < 0.01, Table 2). Similarly, Youden’s index in adults (0.709) was also higher than that in children (0.686, Table 2). These results indicated the diagnostic value of RRS1 expression was better in adults.

**Table 2:** Evaluation of RRS1 expression in children and adults PTC

Gene	Population	AUC	95% CI	*P* value	Youden’s index
RRS1	Children	0.823	(0.649, 0.997)	0.009**	0.686
	Adults	0.871	(0.718, 1.025)	0.001**	0.709
	Total	0.850	(0.701, 0.998)	0.0001***	0.633

** *P* value < 0.01, *** *P* value < 0.001

## Discussion

Here we focus on papillary thyroid carcinoma, one of the most frequent malignancies of the endocrine system, whose mechanisms of transformation are still far from being elucidated [23, 24]. RRS1 has been fully understood in both yeast and plants. But the knowledge of functional human RRS1 is still limited. The role of RRS1 had been found only in Huntington disease by induction of endoplasmic reticulum (ER) stress in neurons for cellular dysfunctions [16]. However, the role of RRS1 has not been reported in cancer. We first analyzed RRS1 silencing in PTC cell line, observing that lower expression of RRS1 significantly decreased TPC-1 cells proliferation. In addition, down-regulation of RRS1 inhibited cell cycle and promoted apoptosis. Meanwhile, silencing of RRS1 led to up-regulation of genes involved in cell death, and caused down-regulation of genes related to blood vessel development. With pathway analysis, genes regulated by RRS1 were also associated with apoptosis, cell proliferation, metabolism and blood vessel development. Our results suggested that RRS1 overexpressed in PTC, which promoted tumorigenesis and accelerated development of blood vessel. Notably, the present study also confirmed the diagnostic value of RRS1 in thyroid carcinoma of both children and adults.

The biological function of human RRS1 in cancer has not been revealed. Our experiments demonstrated that the growth of TPC-1 cell line was significantly impaired after RRS1 silencing. In support with this, our in silico study found that major oncology pathways, like Wnt/β-cateinin signaling pathway and TGF-β signaling pathway, were strongly repressed upon ribosome biogenesis inhibition by RRS1 silencing. Likewise, RRS1 silencing also arrested TPC-1 cells at the G_2_/M phase. To validate these results, we focused on genes related with G_2_/M phase. We found that CCNA2, CDC25C and MAD2L1 were down-regulated, while CDKN1A was up-regulated after RRS1 silencing (Additional file 1: Fig. S4). Besides, the result also showed that RRS1 silencing could increase apoptosis. These results were supported by highly induced gene sets which involved in apoptosis, such as P53 pathway and PTEN pathway. These results also indicated that inhibition of ribosome biogenesis might disrupt oncology pathways thus inhibit cell proliferation and promote apoptosis of PTC. This proposition is consistent with the report that neucleolar protein Nucleostemin promoted cell proliferation of human glioma via Wnt/β-Catenin pathway [25], suggesting abnormal expression of nucleolar protein could active oncology pathway.

Fast proliferating cells have a higher demand for ribosomes. Up-regulation of ribosome production might play an important role in the genesis of cancer [26]. RRS1 is required for ribosome biogenesis in *Saccharomyces cerevisiae* [27–29]. In fact, recent study indicated that human RRS1 existed in nucleolar extracts of HeLa cells [15, 30, 31]. Therefore, RRS1 may participate in PTC progression upon ribosome biogenesis. In agreement with this notion, we found that gene set relative to ribosome biogenesis was significantly repressed. This result is consistent with reports by others that nucleolar protein overexpressed in thyroid tumor cells, including Nucleostemin [32] and B23 [33]. These evidences reinforce the advanced involvement of a disturbed ribosome biogenesis in human papillary thyroid carcinomas.

Beside proliferation and apoptosis of PTC cells, enrichment analysis reveals that aberrant expression of RRS1 may also regulate metabolism and blood vessel development. Firstly, we found that the gene set of glycan degradation was significantly enriched and upregulated upon RRS1 silencing. In line with our result, several studies demonstrated that glycan degradation play key roles in cancer progression and cancer therapy [34]. Moreover, HES/HEY pathway, which play an essential role in blood vessel development and carcinogenesis, was repressed by RRS1 inhibition [35]. In addition, MEF transcription factors, like MEF2D, which is also a key regulator of blood vessel development [36], was repressed by RRS1 inhibition. These results indicated that disrupted ribosomal biogenesis may promote energy metabolism in tumors and inhibit angiogenesis in PTC cells.

Both morphologic features and molecular basis of PTC are different between children and adults. For different genomic basis of PTC among children and adults, we thus supposed that RRS1 expression may relate to age. The results showed that the expression of RRS1 in thyroid carcinoma tissues was higher than that in pericarcinous tissues, and it would be a potential indicator of thyroid carcinoma. Meanwhile, as we expected, higher expression of RRS1 was positively correlated with age (Fig. 5c), meaning lower expression of RRS1 in PTC of children. Therefore, differential expressed RRS1 may be a potential reason of why children PTC patients have more favorable prognosis than adults. In fact, different molecular features of PTC were reported between children and adults. Detection of BRAF and RET/PTC rearrangements is widely used in clinical diagnosis of papillary thyroid carcinoma. Recent molecular studies have shown that BRAF V600E mutations have higher frequency in adult PTC than in children [37, 38]. In contrast, RET/PTC rearrangements are more common among children PTC [11, 39–41]. These studies indicate that PTC in childhood is characterized by a higher prevalence of gene rearrangements and a lower frequency of site mutations compared with that in adults.

Although we found that aberrant expression of RRS1 may accelerate progression of PTC, the limitations of the study should be noted. The precise mechanism of RRS1 in modulating cell cycle process and energy metabolism was not clearly elucidated. Especially, questions like how RRS1 affect cell cycle progression, and what the role of RRS1 is in chemotherapy drug treatment, are interesting. It would be warranted in the future studies to investigate functions of RRS1 accordingly. Besides, due to rare of children PTC, the sample size of children PTC was moderately small. Thus, enlarge the sample size is necessary to evaluate the diagnostic value of RRS1 for papillary thyroid carcinoma both in children and adults.

## Conclusions

This study demonstrated that RRS1 silencing could inhibit cell proliferation, activate G_2_/M phase checkpoint and promote apoptosis in PTC. In addition, silencing RRS1 promoted metabolism, and inhibited development of blood vessel. Our study also provided evidence that PTC had different molecular basis in children and adults. These data afford a comprehensive view of a novel function for human RRS1, who could be a potential indicator of papillary thyroid carcinoma.

## Additional files

**Additional file 1.** Additional figures.

**Additional file 2.** Additional table.

## Abbreviations

PTC: papillary thyroid carcinoma
RRS1: ribosome biogenesis regulator homolog
DTC: differentiated thyroid cancer
MTC: medullary thyroid cancer
LNM: lymph node metastasis
ATA: American Thyroid Association
shRNA: short hairpin RNA
shRNA-NC: shRNA-Negative control
GO: gene ontology
GSEA: gene set enrichment analysis
FDR: false discovery rate
UICC: International Cancer Control
ROC: receiver operating characteristic
IPA: ingenuity pathway analysis
ER: endoplasmic reticulum

## References

[1] Burman KD, Wartofsky L (2015). Clinical practice. Thyroid nodules. N Engl J Med.

[2] Chen W, Zheng R, Baade PD, Zhang S, Zeng H, Bray F, Jemal A, Yu XQ, He J (2016). Cancer statistics in China, 2015. CA Cancer J Clin.

[3] Haugen BR (2017). 2015 American Thyroid Association management guidelines for adult patients with thyroid nodules and differentiated thyroid cancer: what is new and what has changed?. Cancer..

[4] Sherman SI (2003). Thyroid carcinoma. Lancet.

[5] Gupta A, Ly S, Castroneves LA, Frates MC, Benson CB, Feldman HA, Wassner AJ, Smith JR, Marqusee E, Alexander EK (2013). A standardized assessment of thyroid nodules in children confirms higher cancer prevalence than in adults. J Clin Endocrinol Metab.

[6] Francis GL, Waguespack SG, Bauer AJ, Angelos P, Benvenga S, Cerutti JM, Dinauer CA, Hamilton J, Hay ID, Luster M (2015). Management guidelines for children with thyroid nodules and differentiated thyroid cancer. Thyroid.

[7] Jalali-Nadoushan MR, Amirtouri R, Davati A, Askari S, Siadati S (2016). Expression of estrogen and progesterone receptors in papillary thyroid carcinoma. Caspian J Intern Med.

[8] Sinnott B, Ron E, Schneider AB (2010). Exposing the thyroid to radiation: a review of its current extent, risks, and implications. Endocr Rev.

[9] Tan LG, Nan L, Thumboo J, Sundram F, Tan LK (2007). Health-related quality of life in thyroid cancer survivors. Laryngoscope.

[10] Jung CK, Little MP, Lubin JH, Brenner AV, Wells SA, Sigurdson AJ, Nikiforov YE (2014). The increase in thyroid cancer incidence during the last four decades is accompanied by a high frequency of BRAF mutations and a sharp increase in RAS mutations. J Clin Endocrinol Metab.

[11] Fenton CL, Lukes Y, Nicholson D, Dinauer CA, Francis GL, Tuttle RM (2000). The ret/PTC mutations are common in sporadic papillary thyroid carcinoma of children and young adults. J Clin Endocrinol Metab.

[12] Mishra A, Agrawal V, Krishnani N, Mishra SK (2009). Prevalence of RET/PTC expression in papillary thyroid carcinoma and its correlation with prognostic factors in a north Indian population. J Postgrad Med.

[13] Lee J, Seol MY, Jeong S, Lee CR, Ku CR, Kang SW, Jeong JJ, Shin DY, Nam KH, Lee EJ (2015). A metabolic phenotype based on mitochondrial ribosomal protein expression as a predictor of lymph node metastasis in papillary thyroid carcinoma. Medicine (Baltimore).

[14] Miyoshi K, Tsujii R, Yoshida H, Maki Y, Wada A, Matsui Y, Toh EA, Mizuta K (2002). Normal assembly of 60S ribosomal subunits is required for the signaling in response to a secretory defect in *Saccharomyces cerevisiae*. J Biol Chem.

[15] Gambe AE, Matsunaga S, Takata H, Ono-Maniwa R, Baba A, Uchiyama S, Fukui K (2009). A nucleolar protein RRS1 contributes to chromosome congression. FEBS Lett.

[16] Carnemolla A, Fossale E, Agostoni E, Michelazzi S, Calligaris R, De Maso L, Del Sal G, MacDonald ME, Persichetti F (2009). Rrs1 is involved in endoplasmic reticulum stress response in Huntington disease. J Biol Chem.

[17] Sobrinho-Simoes MA, Goncalves V, Sousa-Le F, Cardoso V (1977). A morphometric study of nuclei, nucleoli and nuclear bodies in goitres and papillary thyroid carcinomas. Experientia.

[18] Montironi R, Braccischi A, Scarpelli M, Sisti S, Matera G, Mariuzzi GM, Alberti R, Collan Y (1990). The number of nucleoli in benign and malignant thyroid lesions: a useful diagnostic sign in cytological preparations. Cytopathology.

[19] Zhang J, Harnpicharnchai P, Jakovljevic J, Tang L, Guo Y, Oeffinger M, Rout MP, Hiley SL, Hughes T, Woolford JL (2007). Assembly factors Rpf2 and Rrs1 recruit 5S rRNA and ribosomal proteins rpL5 and rpL11 into nascent ribosomes. Genes Dev.

[20] Mitsiades N, Poulaki V, Tseleni-Balafouta S, Koutras DA, Stamenkovic I (2000). Thyroid carcinoma cells are resistant to FAS-mediated apoptosis but sensitive to tumor necrosis factor-related apoptosis-inducing ligand. Cancer Res.

[21] da Huang W, Sherman BT, Lempicki RA (2009). Bioinformatics enrichment tools: paths toward the comprehensive functional analysis of large gene lists. Nucleic Acids Res.

[22] Subramanian A, Tamayo P, Mootha VK, Mukherjee S, Ebert BL, Gillette MA, Paulovich A, Pomeroy SL, Golub TR, Lander ES, Mesirov JP (2005). Gene set enrichment analysis: a knowledge-based approach for interpreting genome-wide expression profiles. Proc Natl Acad Sci USA.

[23] Nikiforov YE, Nikiforova MN (2011). Molecular genetics and diagnosis of thyroid cancer. Nat Rev Endocrinol.

[24] Chiappetta G, Valentino T, Vitiello M, Pasquinelli R, Monaco M, Palma G, Sepe R, Luciano A, Pallante P, Palmieri D (2015). PATZ1 acts as a tumor suppressor in thyroid cancer via targeting p53-dependent genes involved in EMT and cell migration. Oncotarget.

[25] Bao Z, Wang Y, Yang L, Wang L, Zhu L, Ban N, Fan S, Chen W, Sun J, Shen C, Cui G (2016). Nucleostemin promotes the proliferation of human glioma via Wnt/beta-Catenin pathway. Neuropathology.

[26] Montanaro L, Trere D, Derenzini M (2008). Nucleolus, ribosomes, and cancer. Am J Pathol.

[27] Wan K, Kawara H, Yamamoto T, Kume K, Yabuki Y, Goshima T, Kitamura K, Ueno M, Kanai M, Hirata D (2015). The essential function of Rrs1 in ribosome biogenesis is conserved in budding and fission yeasts. Yeast.

[28] Narusaka M, Hatakeyama K, Shirasu K, Narusaka Y (2014). Arabidopsis dual resistance proteins, both RPS4 and RRS1, are required for resistance to bacterial wilt in transgenic Brassica crops. Plant Signal Behav.

[29] Tsuno A, Miyoshi K, Tsujii R, Miyakawa T, Mizuta K (2000). RRS1, a conserved essential gene, encodes a novel regulatory protein required for ribosome biogenesis in *Saccharomyces cerevisiae*. Mol Cell Biol.

[30] Andersen JS, Lyon CE, Fox AH, Leung AK, Lam YW, Steen H, Mann M, Lamond AI (2002). Directed proteomic analysis of the human nucleolus. Curr Biol.

[31] Scherl A, Coute Y, Deon C, Calle A, Kindbeiter K, Sanchez JC, Greco A, Hochstrasser D, Diaz JJ (2002). Functional proteomic analysis of human nucleolus. Mol Biol Cell.

[32] Pianta A, Puppin C, Passon N, Franzoni A, Romanello M, Tell G, Di Loreto C, Bulotta S, Russo D, Damante G (2011). Nucleophosmin delocalization in thyroid tumour cells. Endocr Pathol.

[33] Onda M, Emi M, Yoshida A, Miyamoto S, Akaishi J, Asaka S, Mizutani K, Shimizu K, Nagahama M, Ito K (2004). Comprehensive gene expression profiling of anaplastic thyroid cancers with cDNA microarray of 25 344 genes. Endocr Relat Cancer.

[34] Taniguchi N, Kizuka Y (2015). Glycans and cancer: role of *N*-glycans in cancer biomarker, progression and metastasis, and therapeutics. Adv Cancer Res.

[35] Zhou M, Yan J, Ma Z, Zhou Y, Abbood NN, Liu J, Su L, Jia H, Guo AY (2012). Comparative and evolutionary analysis of the HES/HEY gene family reveal exon/intron loss and teleost specific duplication events. PLoS ONE.

[36] Sacilotto N, Chouliaras KM, Nikitenko LL, Lu YW, Fritzsche M, Wallace MD, Nornes S, Garcia-Moreno F, Payne S, Bridges E (2016). MEF2 transcription factors are key regulators of sprouting angiogenesis. Genes Dev.

[37] Sobrinho-Simoes M, Maximo V, Rocha AS, Trovisco V, Castro P, Preto A, Lima J, Soares P (2008). Intragenic mutations in thyroid cancer. Endocrinol Metab Clin North Am.

[38] Penko K, Livezey J, Fenton C, Patel A, Nicholson D, Flora M, Oakley K, Tuttle RM, Francis G (2005). BRAF mutations are uncommon in papillary thyroid cancer of young patients. Thyroid.

[39] Demidchik YE, Saenko VA, Yamashita S (2007). Childhood thyroid cancer in Belarus, Russia, and Ukraine after Chernobyl and at present. Arq Bras Endocrinol Metabol.

[40] Monaco SE, Pantanowitz L, Khalbuss WE, Benkovich VA, Ozolek J, Nikiforova MN, Simons JP, Nikiforov YE (2012). Cytomorphological and molecular genetic findings in pediatric thyroid fine-needle aspiration. Cancer Cytopathol.

[41] Yamashita S, Saenko V (2007). Mechanisms of disease: molecular genetics of childhood thyroid cancers. Nat Clin Pract Endocrinol Metab.

