# Supplementary figures and images for: RRS1 gene expression involved in the progression of papillary thyroid carcinoma
Source: Cancer Cell Int. 2018 Feb 13;18:20. doi: 10.1186/s12935-018-0519-x (PMC5812111; doi:10.1186/s12935-018-0519-x)

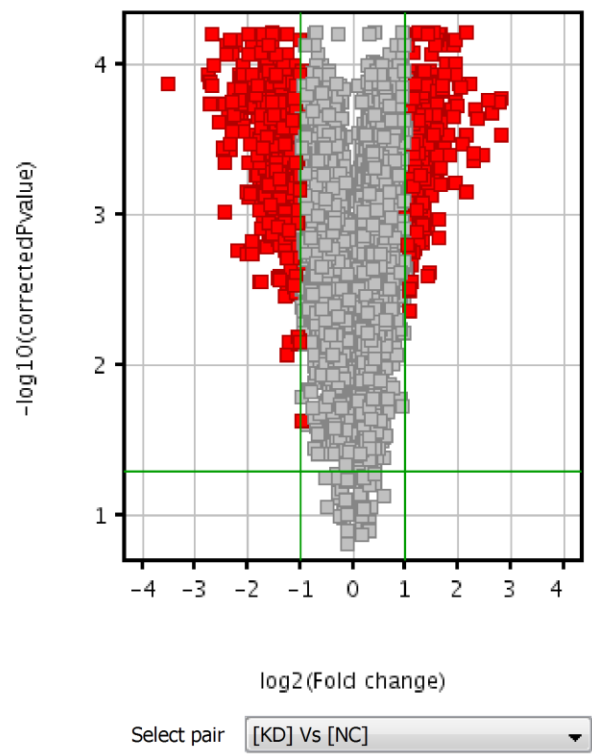

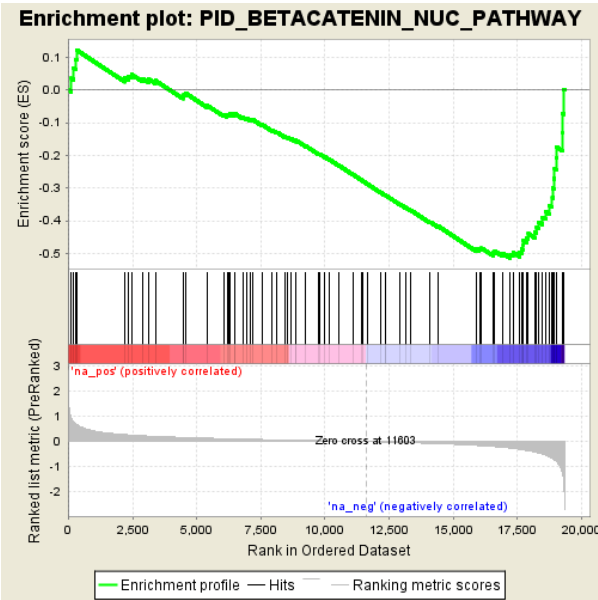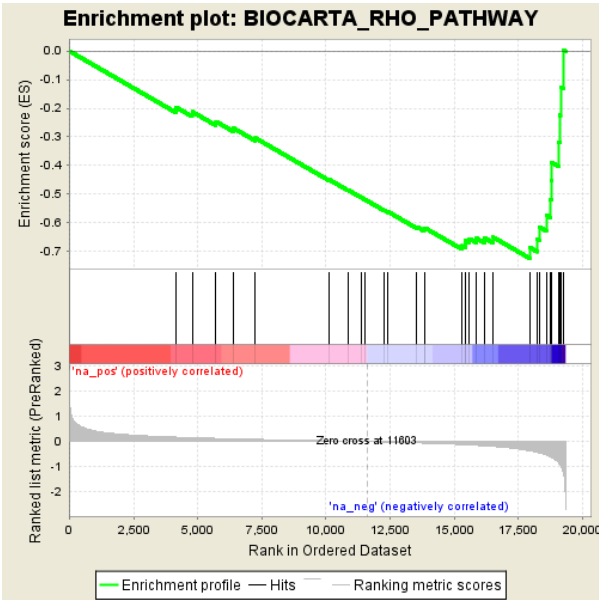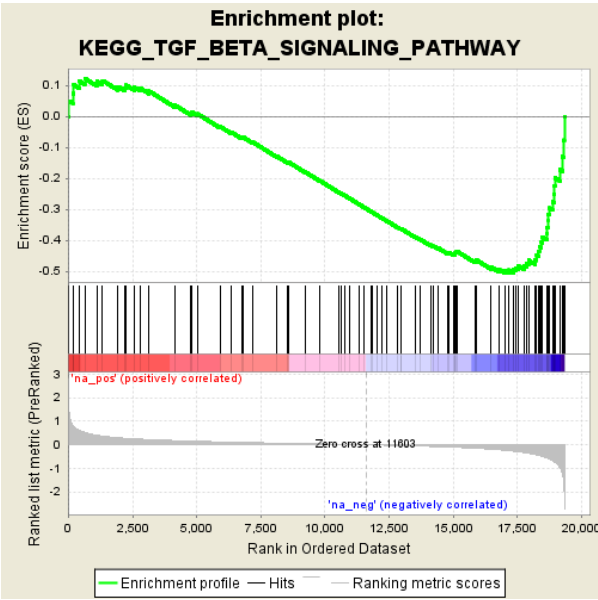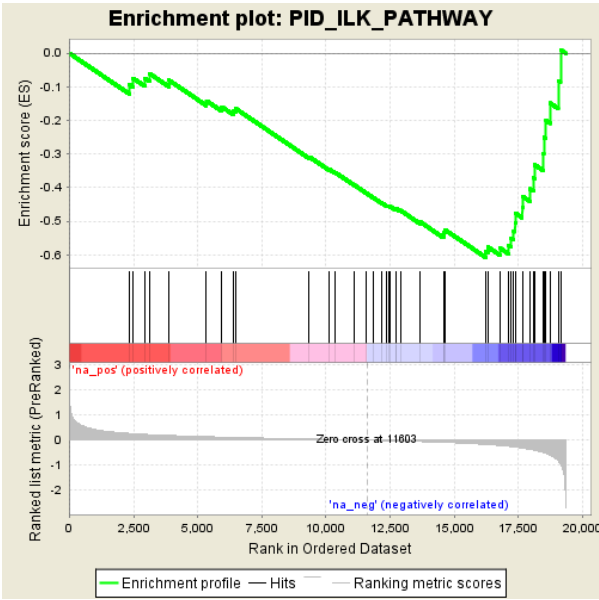

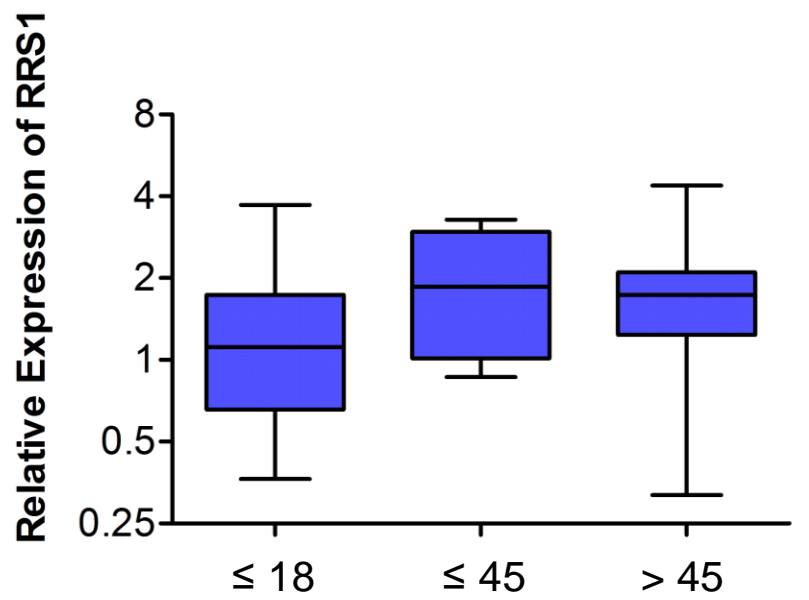

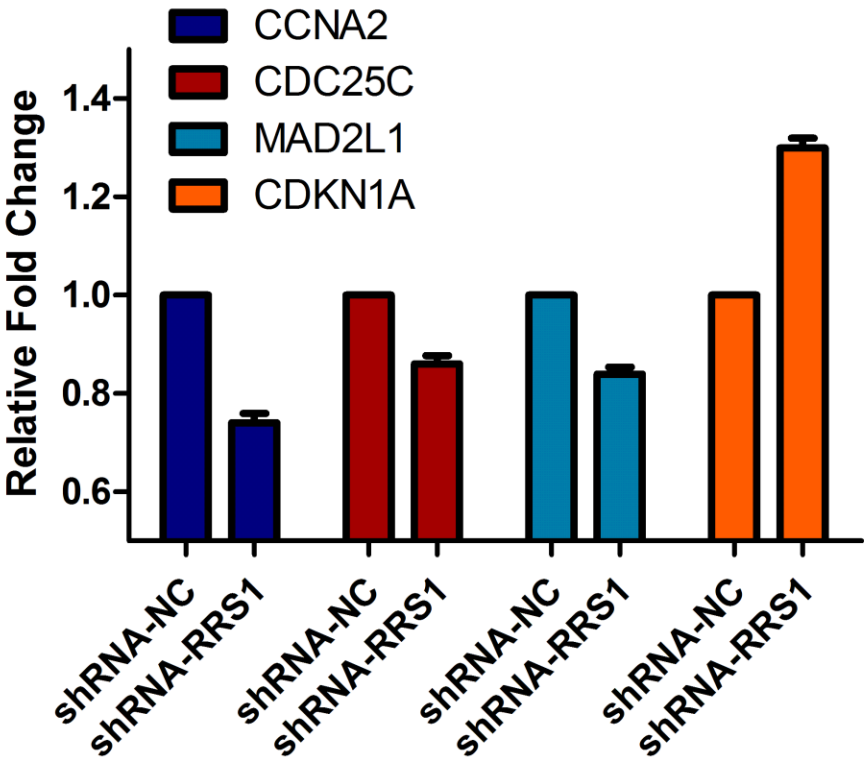

Supplement: Supplementary file 1 — Additional file 1. Additional figures. [file 12935_2018_519_MOESM1_ESM.pdf]
